# Sutureless Aortic Valve Replacement *vs.* Transcatheter Aortic Valve Implantation in Patients with Small Aortic Annulus: Clinical and Hemodynamic Outcomes from a Multi-Institutional Study

**DOI:** 10.21470/1678-9741-2023-0155

**Published:** 2024-07-15

**Authors:** Lorenzo Di Bacco, Michele D’Alonzo, Marco Di Eusanio, Fabrizio Rosati, Marco Solinas, Massimo Baudo, Thierry Folliguet, Stefano Benussi, Theodor Fischlein, Claudio Muneretto

**Affiliations:** 1 Department of Cardiac Surgery, ASST Spedali Civili di Brescia, University of Brescia, Brescia, Italy; 2 Cardiovascular Surgery Department, Ospedali Riuniti, Ancona, Italy; 3 Department of Adult Cardiac Surgery, G. Pasquinucci Heart Hospital, Massa, Italy; 4 Cardiac surgery, Centre Hospitalier Universitaire Mondor, Créteil, France; 5 Department of Cardiac Surgery, Klinikum Nürnberg, Nuremberg, Germany

**Keywords:** Transcatheter Aortic Valve Replacement, Aorta Valve, Hemodynamics, Prostheses and Implants, Propensity Scores

## Abstract

**Objective:**

This study aimed to compare hemodynamic performances and clinical outcomes of patients with small aortic annulus (SAA) who underwent aortic valve replacement by means of sutureless aortic valve replacement (SUAVR) or transcatheter aortic valve implantation (TAVI).

**Methods:**

From 2015 to 2020, 622 consecutive patients with SAA underwent either SUAVR or TAVI. Through a 1:1 propensity score matching analysis, two homogeneous groups of 146 patients were formed. Primary endpoint: all cause-death at 36 months. Secondary endpoints: incidence of moderate to severe patient-prosthesis mismatch (PPM) and incidence of major adverse cardiovascular and cerebrovascular events (MACCEs)

**Results:**

All-cause death at three years was higher in the TAVI group (SUAVR 12.2% *vs.* TAVI 21.0%, *P*=0.058). Perioperatively, comparable hemodynamic performances were recorded in terms of indexed effective orifice area (SUAVR 1.12 ± 0.23 cm^2^/m^2^ *vs.* TAVI 1.17 ± 0.28 cm^2^/m^2^, *P*=0.265), mean transvalvular gradients (SUAVR 12.9 ± 5.3 mmHg *vs.* TAVI 12.2 ± 6.2 mmHg, *P*=0.332), and moderate-to-severe PPM (SUAVR 4.1% *vs.* TAVI 8.9%, *P*=0.096). TAVI group showed a higher cumulative incidence of MACCEs at 36 months (SUAVR 18.1% *vs.* TAVI 32.6%, *P*<0.001). Pacemaker implantation (PMI) and perivalvular leak ≥ 2 were significantly higher in TAVI group and identified as independent predictors of mortality (PMI: hazard ratio [HR] 3.05, 95% confidence interval [CI] 1.34-6.94, *P*=0.008; PPM: HR 2.72, 95% CI 1.25-5.94, *P*=0.012).

**Conclusion:**

In patients with SAA, SUAVR and TAVI showed comparable hemodynamic performances. Moreover, all-cause death and incidence of MACCEs at follow-up were significantly higher in TAVI group.

## INTRODUCTION

Small aortic annulus (SAA) is an anatomic feature that represents an important concern in patients undergoing aortic valve replacement (AVR)^[1]^. Small sizes (≤ 23 mm) of stented aortic bioprostheses have an effective orifice area (EOA) smaller than the native aortic valve area, which may lead to patient-prosthesis mismatch (PPM)^[1,2]^. As a matter of fact, PPM occurs when the EOA of a normally functioning prosthetic valve is too small in relation to the patient’s body surface^[2]^.

The presence of moderate (< 0.85 cm^2^/m^2^ and > 0.65 cm^2^) or severe (< 0.65 cm^2^/m^2^) PPM has been demonstrated to produce detrimental effects on patients’ outcomes, jeopardizing left ventricular reverse remodeling, hypertrophy regression, and functional recovery^[1,3]^.

Surgical aortic annulus enlargement was demonstrated to be a viable surgical strategy to reduce PPM rate, allowing surgeons to implant larger bioprostheses. However, aortic annulus enlargement increases surgical complexity and risks and is rarely performed^[4]^. Nevertheless, the use of stentless bioprostheses (SB) reduced the risk of PPM in patients with SAA since the absence of a rigid stent allows the use of larger prostheses. However, the major drawback of SB is the increased ischemic and cardiopulmonary bypass (CPB) times for implant, despite no differences in terms of intensive care unit (ICU) and hospital length of stay were demonstrated^[5,6]^. Several studies showed that transcatheter aortic valve implantation (TAVI) offered better hemodynamic results with a reduced incidence of PPM especially in patients with a SAA^[7,8]^. In this specific subset of population, self-expandable valves (SEV) showed better hemodynamic performances when compared to balloon-expandable valves (BEV)^[9,10]^.

Sutureless aortic valves proved to have larger EOAs for any given size compared to stented bioprostheses and to provide good hemodynamic performances, comparable to stentless valves. In addition, sutureless valves can be implanted with significantly shorter aortic cross-clamping and CPB times, overcoming the drawback of SB^[11]^. Patients receiving sutureless valves had shorter invasive ventilation time and ICU and hospital stay as well as the need for red blood cell transfusions when compared to stented valves^[11]^.

The aim of this study was to compare hemodynamic performances and outcomes of sutureless aortic valve replacement (SUAVR) *vs.* TAVI in elderly patients affected by aortic stenosis (AS) with a small aorta undergoing surgical AVR employing balloon-expandable or self-expandable bioprostheses.

## METHODS

### Study Design

This European multi-institutional retrospective study included patients with a SAA (echocardiographic diameter ≤ 21 mm) who underwent AVR by means of either surgical SUAVR or TAVI for isolated AS.

The study protocol was approved by the Institutional Review Board of each participating center (University of Brescia approved the present study with NP 1870). Data were collected from May 2015 to December 2020 from five European centers. A total of 320 and 302 patients with a SAA were recruited for the SUAVR and TAVI groups, respectively.

A propensity score matching analysis was performed to reduce selection bias. Following 1:1 propensity score matching, 146 patients from each treatment group were selected to obtain two homogeneous populations.

Patients in the surgical group were treated with Perceval® S valve (LivaNova PLC, London, United Kingdom) size S (19-21 mm) or M (21-23 mm), while patients in the TAVI group were treated with either SAPIEN XT®/SAPIEN 3® (Edwards Lifesciences, Irvine, California, United States of America) size 23, CoreValve™/Evolut™ R (Medronic, Minneapolis, Minnesota, United States of America) size 23 or 26, or Acurate TA™ (Symetys SA, Ecublens, Switzerland) size S. Transthoracic echocardiography was performed at baseline, at discharge, and at the first and third years postoperatively in all patients. Moderate to severe PPM was defined as indexed EOA (iEOA) (moderate PPM iEOA < 0.85 cm^2^/m^2^; severe PPM iEOA < 0.65 cm^2^/m^2^)^[1]^. Transesophageal echocardiography was performed to assess intraoperative implant success according to Valve Academic Research Consortium (VARC) III criteria^[12]^. Prosthetic aortic valve regurgitation was defined moderate to severe according to VARC III criteria (vena contracta > 4 mm, pressure half-time 200-500 ms, regurgitant volume > 30 ml/beat)^[12]^.

As far as TAVI concerns, oversizing was analyzed by the physicians involved in the individual case and did not exceed 20%. For sutureless valves, oversizing was not performed, as recommended in the Company’s manual.

### Study Endpoints

The primary endpoints of the study were all-cause mortality and hemodynamic valve performances (mean/peak gradients, EOA, iEOA, moderate-severe PPM). Secondary endpoints included major adverse cardiovascular and cerebrovascular events (MACCEs) defined as follows: all-cause death, stroke/transitory ischemic attack (TIA), endocarditis, reoperation, pacemaker implantation (PMI), and perivalvular leak (PVL) ≥ 2.

### Statistical Analysis

The normality of continuous distributions was assessed using the Kolmogorov-Smirnov test. Normally and skewed distributed variables were presented as mean with standard deviation and median with 25^th^ and 75^th^ percentiles (interquartile range boundaries), respectively. Student’s *t*-test or Mann-Whitney U test were used for normally distributed or skewed distributed variables, respectively. Categorical variables were expressed as frequency and percentage and were compared using the Chi-square test.

**Fig. 1:**
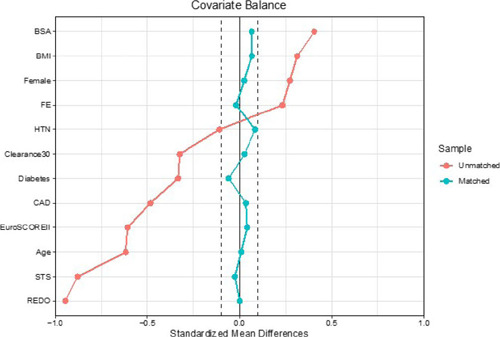
Propensity score Love plot. BMI=body mass index; BSA=body surface area; CAD=coronary artery disease; EuroSCORE=European System for Cardiac Operative Risk Evaluation; EF=ejection fraction; HTN=hypertension; STS=Society of Thoracic Surgeons.

Preoperative covariates were adjusted with 1:1 nearest-neighbour propensity score matching without replacement (caliper 0.06), obtaining two balanced groups (matched [Table 1] and unmatched [Table E1]). Balance check was performed analyzing the standard mean difference between the two groups. A visual inspection of the standard mean difference with the Love plot was also performed. The matched standardized differences of each covariate in the matched population were < 10% (Figure 1).

**Table 1:** Patients’ preoperative characteristics.

	Unmatched			Matched		
	TAVI	Perceval®	*P*-value	TAVI	Perceval®	*P*-value
	(n=302)	(n=320)		(n=146)	(n=146)	
Age (years)	83.23 ± 5.58	79.63 ± 5.68	< 0.001	81.14 ± 6.01	81.19 ± 5.29	0.946
BMI (kg/m²) (mean ± SD)	25.81 ± 4.96	25.15 ± 5.07	< 0.001	24.9 ± 5.27	24.9 ± 5.08	0.623
BSA (m²) (mean ± SD)	1.63 ± 0.28	1.60 ± 0.16	< 0.001	1.58 ± 0.19	1.57 ± 0.18	0.619
Females	274 (90.7%)	291 (90.9%)	< 0.001	129 (88.4%)	130 (89.0%)	0.853
STS risk score (mean ± SD)	8.08 ± 5.21	4.93 ± 3.82	< 0.001	6.14 ± 3.93	6.04 ± 4.66	0.838
EuroSCORE II (mean ± SD)	7.91 ± 5.48	5.27 ± 4.56	< 0.001	5.47 ± 4.02	5.65 ± 4.86	0.729
Redo	46 (15.2%)	17 (5.3%)	< 0.001	15 (10.3%)	14 (9.6%)	0.845
Hypertension	298 (98.7%)	255 (79.7%)	0.002	119 (81.5%)	124 (84.9%)	0.287
Dyslipidemia	172 (57.0%)	155 (48.4%)	0.483	76 (52.1%)	69 (47.3%)	0.483
Diabetes	149 (49.3%)	87 (27.2%)	< 0.001	61 (41.8%)	54 (37.0%)	0.402
COPD	33 (10.9%)	47 (14.7%)	0.057	17 (11.6%)	22 (15.1%)	0.377
Clearance < 30	55 (18.2%)	19 (5.9%)	< 0.001	19 (13.0%)	20 (13.7%)	0.863
CAD	138 (45.7%)	70 (21.9%)	< 0.001	48 (32.9%)	48 (32.9%)	0.999
PAD	32 (10.6%)	37 (11.6%)	0.391	9 (6.2%)	18 (12.3%)	0.069
CVA (previous)	28 (9.3%)	15 (4.7%)	0.059	11 (7.5%)	9 (6.2%)	0.643
Ejection fraction (mean ± SD)	57.6 ± 10.8	60.2 ± 10.3	0.014	58.7 ± 10.6	58.5 ± 10.7	0.854
**Preoperative echocardiography**						
Gmax (mean ± SD)	80.8 ± 20.2	82.4 ± 25.5	0.813	82.2 ± 19.8	80.2 ± 22.5	0.407
Gmean (mean ± SD)	50.1 ± 13.8	50.3 ± 16.6	0.616	51.8 ± 13.3	50.8 ± 16.1	0.555
Effective orifice area (cm²) (mean ± SD)	0.64 ± 0.2	0.65 ± 0.21	0.361	0.63 ± 0.20	0.64 ± 0.21	0.635
Mean aortic annulus (mm) (mean ± SD)	20.4 ± 0.5	20.3 ± 0.6	0.853	20.3 ± 0.7	20.2 ± 0.8	0.733

BMI=body mass index; BSA=body surface area; CAD=coronary artery disease; COPD=chronic obstructive pulmonary disease; CVA=cerebrovascular accident; EuroSCORE=European System for Cardiac Operative Risk Evaluation; Gmax=maximum gradient; Gmean=mean gradient; PAD=peripheral artery disease; SD=standard deviation; STS=Society of Thoracic Surgeons; TAVI=transcatheter aortic valve implantation

**Table E1:** Preoperative patients’ characteristics in unmatched groups.

	Unmatched		
	TAVI	Perceval®	*P*-value
	(n = 302)	(n = 320)	
Age (years)	83.23 ± 5.58	79.63 ± 5.68	< 0.001
BMI (kg/m²) (mean ± SD)	25.81 ± 4.96	25.15 ± 5.07	< 0.001
BSA (m²)(mean ± SD)	1.63 ± 0.28	1.60 ± 0.16	< 0.001
Female sex	274 (90.7%)	291 (90.9%)	< 0.001
STS risk score (mean ± SD)	8.08 ± 5.21	4.93 ± 3.82	< 0.001
EuroSCORE II (mean ± SD)	7.91 ± 5.48	5.27 ± 4.56	< 0.001
Redo	46 (15.2%)	17 (5.3%)	< 0.001
Hypertension	298 (98.7%)	255 (79.7%)	0.002
Dyslipedemia	172 (57.0%)	155 (48.4%)	0.483
Diabetes	149 (49.3%)	87 (27.2%)	< 0.001
COPD	33 (10.9%)	47 (14.7%)	0.057
Clearance < 30	55 (18.2%)	19 (5.9%)	< 0.001
CAD	138 (45.7%)	70 (21.9%)	< 0.001
PAD	32 (10.6%)	37 (11.6%)	0.391
CVA (previous)	28 (9.3%)	15 (4.7%)	0.059
Ejection fraction (mean ± SD)	57.6 ± 10.8	60.2 ± 10.3	0.014
*Preoperative echocardiography*			
Gmax (mean ± SD)	80.8 ± 20.2	82.4 ± 25.5	0.813
Gmean (mean ± SD)	50.1 ± 13.8	50.3 ± 16.6	0.616
Effective orifice area, cm² (mean ± SD)	0.64 ± 0.2	0.65 ± 0.21	0.361
Mean aortic annulus (mm) (mean ± SD)	20.4±0.5	20.3±0.6	0.853

BMI=body mass index; BSA=body surface area; CAD=coronary artery disease; COPD=chronic obstructive pulmonary disease; CVA=cerebrovascular accident; EuroSCORE=European System for Cardiac Operative Risk Evaluation; Gmax=maximum gradient; Gmean=mean gradient; PAD=peripheral artery disease; SD=standard deviation; STS=Society of Thoracic Surgeons; TAVI=transcatheter aortic valve implantation

The Kaplan-Meier method was used to assess overall survival and freedom from MACCE. Group difference analysis was evaluated using the log-rank test. A univariate and multivariate Cox-regression analysis was performed to further assess late mortality. Follow-up information was completed by patient or physician contact.

Microsoft® Office Excel 365 software (Microsoft, Redmond, Washington) was used for data extraction and statistical analyses were conducted applying IBM Corp. Released 2017, IBM SPSS Statistics for MAC, version 25.0, Armonk, NY: IBM Corp. and R Project for Statistical Computing, version 3.6.2, using the “MatchIt” package.

## RESULTS

### Operative Results

In the SUAVR group, a minimally invasive strategy was adopted in 60.2% of patients (ministernotomy 52.7%, right anterior thoracotomy 7.5%); in the remaining patients, a median sternotomy was performed. Furthermore, Perceval® S size S was used in 84 (57.6%) patients while size M valve was implanted in 62 (43.4%) patients.

Among TAVI patients, SAPIEN 3® or SAPIEN XT® BEV N. 23 was used in 109 (109/146 [74.6%]) patients, 26 (17.8%) patients had Evolut™ R/CoreValve™ SEV (size 23: 18 patients; size 26: eight patients), and 11 (7.6%) patients had a size S Acurate TA™ self-expandable bioprosthesis. Moreover, in 71.9% of patients, TAVI procedure was carried out through transfemoral (TF) approach, while transapical (TA) approach was adopted in 26.0% of cases, and subclavian, transaortic, and transcarotid approaches in 2.0% of the remaining cases.

A second valve implantation was required for technical failure in three (2.0%) patients in the TAVI group (two patients undergoing Edwards SAPIEN® and one patient receiving an Evolut™ R valve). Emergency conversion to open surgery was required during three (2.0%) procedures: left coronary ostium obstruction and for aortic annular rupture occurred in one (0.7%) and two (1.4%) patients, respectively. In the SUAVR group, one patient was converted to stented valve implantation due to intraoperative annular rupture, while one patient required a second cross-clamp for valve repositioning (Table E2).

**Table E2:** Intraoperative patients’ characteristics.

	Unmatched			Matched		
	TAVI	Perceval®	*P*-value	TAVI	Perceval®	*P*-value
	(n = 302)	(n = 320)		(n=146)	(n=146)	
Non elective procedures	11 (3.64%)	4 (1.25%)	0.056	6 (4.1)	2 (1.4%)	0.151
MAV > 48 hours	10 (3.3%)	8 (2.5%)	0.709	5 (3.4%)	5 (3.4%)	1'000
ICU stay, hours (median, IQR)	21 (18-24)	22 (19-24)	0.144	20 (18-24)	21 (18-24)	0.283
Valve diameter (median, IQR)	23 (23-23)			23 (23-23)		
CPB time (min) (mean ± SD)		65.2 ± 28.7			61.2 ± 25.7	
Aortic cross-clamping time (min) (mean ± SD)		42.5 ± 19.7			39.9 ± 18.8	
*Perceval® size*						
Size S		198 (61.8%)			84 (57.6%)	
Size M		122 (28.2%)			62 (42.4%)	
*Surgical approach*						
Sternotomy		107 (33.4%)			58 (39.7%)	
Ministernotmy		194 (60.6%)			77 (52.7)	
Anterior minithoracotomy		19 (6.0%)			11 (7.5%)	
*TAVI approach*						
Transapical	90 (29.8%)			38 (26.0%)		
Transfemoral	198 (65.5%)			105 (71.9%)		
Other transvessel	14 (4.7%)			3 (2.1%)		

CPB=cardiopulmonary bypass; ICU=intensive care unit; IQR=interquartile range; MAV=mechanical invasive ventilation; SD=standard deviation; TAVI=transcatheter aortic valve implantation

### Early Postoperative Results

**Fig. 2:**
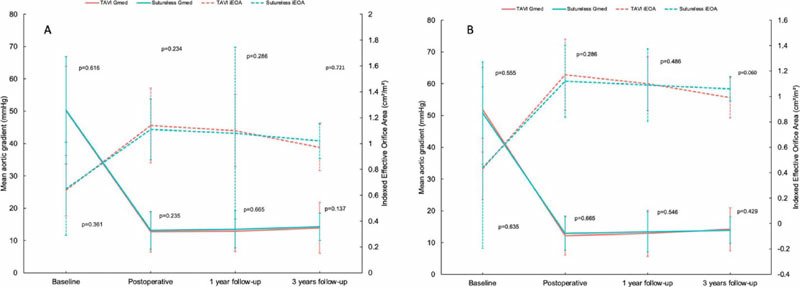
A) Mean gradient (Gmean) and indexed effective orifice area (iEOA) at baseline, discharge, 1 year, and 3 years in unmatched population. B) Mean gradient and iEOA at baseline, discharge, 1 year, and 3 years in matched population. TAVI=transcatheter aortic valve implantation.

Postoperative echocardiography at discharge showed comparable mean gradients between groups (matched: SUAVR 12.9 ± 5.33 mmHg; TAVI 12.16 ± 6.24 mmHg, *P*=0.523), as well as comparable postoperative iEOA (matched: SUAVR 1.12 ± 0.13 cm^2^/m^2^; TAVI 1.17 ± 0.31 cm^2^/m^2^, *P*=0.798) (Figure 2). No differences were reported in terms of postoperative moderate to severe PPM between SUAVR and TAVI (matched: SUAVR 4.1% *vs.* TAVI 8.9%, *P*=0.096). Moreover, no differences were reported between BEV and SEV TAVI in terms of PPM (matched: BEV 10.1% *vs.* SEV 5.2%, *P*=0.391).

Thirty-day all-cause mortality was higher in the TAVI group (matched: SUAVR 1.4% *vs.* TAVI 6.2%, *P*<0.032). Of note, as a subgroup analysis, TA group showed a higher mortality rate compared to TF approach (TA: 13.2% *vs.* TF: 3.7%, *P*=0.03), while no difference in terms of 30-day mortality rate is reported between BEV (5.5%) and SEV (8.8%) (*P*=0.569).

Cumulative incidence of MACCE at 30-days was superior in the TAVI group (matched: SUAVR 10.2% *vs.* TAVI 18.4%, *P*=0.045). On this regard, a higher incidence of atrioventricular (AV) blocks requiring PMI occurred in the TAVI group, both in the matched and unmatched population (matched: SUAVR 4.79% *vs.* TAVI 11.64%, *P*=0.033; unmatched: SUAVR 6.9% *vs.* TAVI 12.2, *P*=0.022), as well as a higher incidence of PVL ≥ 2 was reported in the TAVI group (matched: SUAVR 1.4% *vs.* TAVI 6.8%, *P*=0.017). Moreover, the TAVI group had a significantly higher rate of vascular complications requiring surgical or endovascular interventions (matched: SUAVR 0.68% *vs.* TAVI 9.59%, *P*<0.001).

There were no significant differences between the groups in terms of incidence of stroke/TIA (matched: SUAVR 0.7% *vs.* TAVI 2.6%, *P*=0.370) and acute renal failure (matched: SUAVR 3.5% *vs.* TAVI 7.5%, *P*=0.122).

A superior rate of postoperative transfusions was accounted in the SUAVR group (matched: SUAVR 24.6% *vs.* TAVI 2.7%, *P*<0.001). Conversely, higher incidences of infections requiring antibiotic therapy were reported in the TAVI group (unmatched: SUAVR 3.75% *vs.* TAVI 8.28%, *P*=0.017), however this was not significant after propensity matching (matched: SUAVR 1.4% *vs.* TAVI 4.8%, *P*=0.172). Postoperative results are listed in Table 2 for the matched and in Table E3 for the unmatched groups.

**Table 2:** Postoperative outcomes.

	Unmatched			Matched		
	TAVI	Perceval®	*P*-value	TAVI	Perceval®	*P*-value
	(n=302)	(n=320)		(n=146)	(n=146)	
30-day all-cause mortality	26 (8.61%)	4 (1.3%)	< 0.001	9 (6.1)	2 (1.4%)	0.032
Permanent PM implantation	37 (12.2%)	22 (6.9%)	0.022	17 (11.6%)	7 (4.8%)	0.033
Red blood cell transfusion	11 (3.6%)	75 (23.4%)	< 0.001	4 (2.7%)	36 (24.6%)	< 0.001
Life-threatening bleeding	5 (1.66%)	12 (3.8%)	0.109	2 (1.4%)	4 (1.4%)	0.409
Acute renal failure (stage 2-3, VARC III)	16 (5.3%)	8 (2.5%)	0.071	11 (7.5%)	5 (3.5%)	0.122
Infections requiring antibiotic therapy	25 (8.3%)	13 (4.0%)	0.021	2 (1.4%)	7 (4.8%)	0.172
Vascular complications	24 (7.9%)	5 (1.6%)	< 0.001	14 (9.6%)	2 (1.4%)	< 0.001
Stroke/TIA	11 (3.6%)	7 (2.2%)	0.279	4 (2.7%)	1 (0.7%)	0.370
Myocardial infarction	5 (1.66%)	3 (0.9%)	0.426	3 (2.1%)	1 (0.7%)	0.313
**Postoperative Echocardiography**						
Gmax (mean ± SD)	21.8 ± 8.3	23.1 ± 8.1	0.628	22.5 ± 8.4	23.2 ± 9.2	0.523
Gmean (mean ± SD)	12.7 ± 6.3	13.1 ± 5.8	0.234	12.2 ± 6.2	12.9 ± 5.3	0.265
Effective orifice area (cm²) (mean ± SD)	1.59 ± 0.27	1.51 ± 0.21	0.342	1.63 ± 0.26	1.55 ± 0.15	0.286
iEOA (cm²/m²) (mean ± SD)	1.14 ± 0.29	1.11 ± 0.13	0.235	1.17 ± 0.28	1.12 ± 0.23	0.337
PVL ≥ grade II	16 (5.3%)	7 (2.2%)	0.039	10 (6.8%)	2 (1.4%)	0.017
Moderate to severe PPM	23 (7.6%)	15 (4.6%)	0.127	13 (8.9%)	6 (4.1%)	0.096
**1-year Echocardiography**						
Gmax (mean ± SD)	21.8 ± 8.3	23.1 ± 8.1	0.628	23.5 ± 8.4	24.2 ± 9.2	0.523
Gmean (mean ± SD)	12.9 ± 6.3	13.5 ± 5.8	0.286	12.9 ± 6.2	13.4 ± 5.3	0.265
Effective orifice area (cm²) (mean ± SD)	1.49 ± 0.24	1.45 ± 0.21	0.632	1.52 ± 0.22	1.50 ± 0.19	0.486
iEOA (cm²/m²) (mean ± SD)	1.10 ± 0.28	1.08 ± 0.13	0.665	1.11 ± 0.21	1.09 ± 0.23	0.537
PVL ≥ grade II	23 (7.6%)	14 (4.3%)	0.050	13 (8.2%)	5 (3.4%)	0.050
Moderate to severe PPM	28 (9.2%)	21 (6.5%)	0.127	15 (10.2%)	7 (4.7%)	0.078
**3-year Echocardiography**						
Gmax (mean ± SD)	25.5 ± 7.3	24.7 ± 8.2	0.423	26.5 ± 7.3	25.8 ± 8.2	0.632
Gmean (mean ± SD)	13.9 ± 7.9	14.3 ± 4.2	0.721	14.2 ± 6.8	13.9 ± 4.2	0.429
Effective orifice area (cm²) (mean ± SD)	1.30 ± 0.26	1.39 ± 0.15	0.027	1.33 ± 0.26	1.41 ± 0.15	0.096
iEOA (cm²/m²) (mean ± SD)	0.97 ± 0.18	1.02 ± 0.21	0.137	0.99 ± 0.19	1.06 ± 0.15	0.057
PVL ≥ grade II	36 (11.9%)	24 (7.5%)	0.058	20 (13.9%)	9 (6.1%)	0.031
Moderate to severe PPM	41 (13.5%)	30 (9.3%)	0.098	23 (15.7%)	12 (8.2%)	0.047

Gmax=maximum gradient; Gmean=mean gradient; iEOA=indexed effective orifice area; PM=pacemaker; PPM=patient-prosthesis mismatch; PVL=perivalvular leak; SD=standard deviation; TAVI=transcatheter aortic valve implantation; TIA=transitory ischemic attack; VARC=Valve Academic Research Consortium

**Table E3:** Postoperative outcomes in unmatched groups.

	Unmatched		
	TAVI	Perceval®	*P*-value
	(n = 302)	(n = 320)	
30-day all-cause mortality	26 (8.61%)	4 (1.3%)	< 0.001
Permanent PM implantation	37 (12.2%)	22 (6.9%)	0.022
Red blood cell transfusion	11 (3.6%)	75 (23.4%)	< 0.001
Life-treatening bleeding	5 (1.66%)	12 (3.8%)	0.109
Acute renal failure (stage 2-3, VARC III)	16 (5.3%)	8 (2.5%)	0.071
Infections requiring antibiotic therapy	25 (8.3%)	13 (4.0%)	0.021
Vascular complications	24 (7.9%)	5 (1.6%)	< 0.001
Stroke/TIA	11 (3.6%)	7 (2.2%)	0.279
Myocardial Infarction	5 (1.66%)	3 (0.9%)	0.426
*Postoperative echocardiography*			
Gmax (mean ± SD)	21.8 ± 8.3	23.1 ± 8.1	0.628
Gmean (mean ± SD)	12.7 ± 6.3	13.1 ± 5.8	0.234
Effective orifice area, cm² (mean ± SD)	1.59 ± 0.27	1.51 ± 0.21	0.342
EOA index, cm²/m² (mean ± SD)	1.14 ± 0.29	1.11 ± 0.13	0.235
PVL ≥ grade II	16 (5.3%)	7 (2.2%)	0.039
Moderate to severe PPM	23 (7.6%)	15 (4.6%)	0.127
*1-year echocardiography*			
Gmax (mean ± SD)	21.8 ± 8.3	23.1 ± 8.1	0.628
Gmean (mean ± SD)	12.9 ± 6.3	13.5 ± 5.8	0.286
Effective orifice area, cm² (mean ± SD)	1.49 ± 0.24	1.45 ± 0.21	0.632
EOA index, cm²/m² (mean ± SD)	1.10 ± 0.28	1.08 ± 0.13	0.665
PVL ≥ grade II	23 (7.6%)	14 (4.3%)	0.050
Moderate to severe PPM	28 (9.2%)	21 (6.5%)	0.127
*3-year echocardiography*			
Gmax (mean ± SD)	25.5 ± 7.3	24.7 ± 8.2	0.423
Gmean (mean ± SD)	13.9 ± 7.9	14.3 ± 4.2	0.721
Effective orifice area, cm² (mean ± SD)	1.30 ± 0.26	1.39 ± 0.15	0.027
EOA index, cm²/m² (mean ± SD)	0.97 ± 0.18	1.02 ± 0.21	0.137
PVL ≥ grade II	36 (11.9%)	24 (7.5%)	0.058
Moderate to severe PPM	41 (13.5%)	30 (9.3%)	0.098

EOA=effective orifice area; Gmax=maximum gradient; Gmean=mean gradient; PM=pacemaker; PPM=patient-prosthesis mismatch; PVL=perivalvular leak; SD=standard deviation; TAVI=transcatheter aortic valve implantation; TIA=transitory ischemic attack; VARC=Valve Academic Research Consortium

### Follow-up Results

**Fig. 3:**
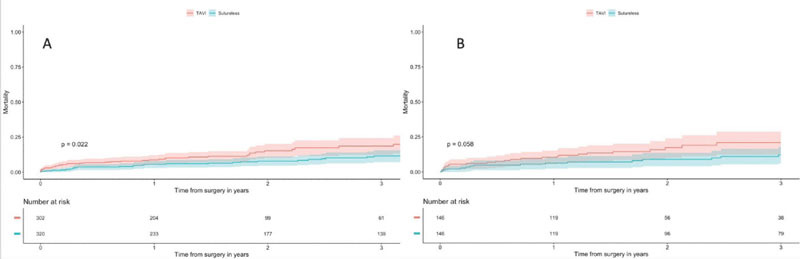
A) All-cause death Kaplan-Meier curves (unmatched groups). B) All-cause death Kaplan-Meier curves (matched groups). TAVI=transcatheter aortic valve implantation.

Mean follow-up was 24.4 ± 11.1 months. All-cause death was significantly higher in the TAVI group at 36 months in the unmatched population (36 months: SUAVR 11.5%, 95% confidence interval [CI] 7.6-15.6%; TAVI 19.9%, 95% CI 13.1-26.2%, *P*=0.022) (Figure 3A), and close to be significant in the matched population (36 months: SUAVR 12.2%, 95% CI 6.1-17.9%; TAVI 21.0%, 95% CI 12.3-28.8%, *P*=0.058) (Figure 3B).

**Supplementary Fig. 1:**
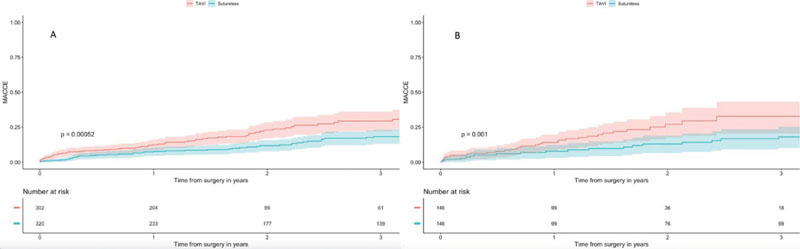
Comparison of patient-prosthesis mismatch (PPM) at discharge, 1 year, and 3 years between sutureless aortic valve replacement (SUAVR) and transcatheter aortic valve implantation (TAVI) in unmatched (A) and matched (B) populations.

At 36 months, a significantly higher incidence of moderate to severe PPM and PVL occurred in the TAVI group when compared to SUAVR (PPM matched: SUAVR 8.2% *vs.* TAVI 15.7%, *P*=0.047; PVL matched: SUAVR 6.1% *vs.* TAVI 13.9%, *P*=0.031) (Table 2, Supplementary Figure 1).

The multivariable Cox regression analysis (time-dependent variable) showed PMI and PPM as independent predictors of death (PMI hazard ratio [HR] 3.05, 95% CI 1.34-6.94, *P*=0.008; PPM HR 2.72, 95% CI 1.25-5.94, *P*=0.012).

**Fig. 4:**
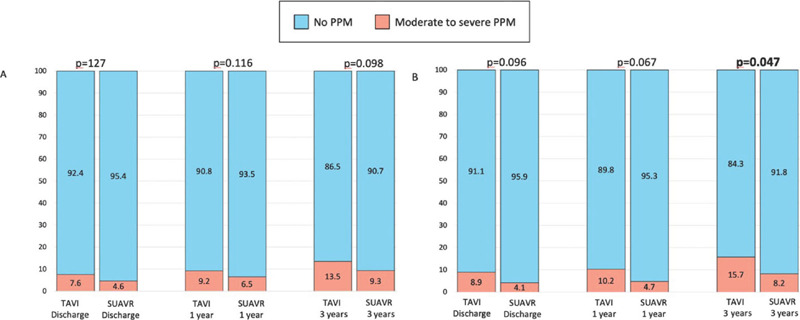
A) Major adverse cardiovascular and cerebrovascular event (MACCE) incidence, Kaplan-Meier curves (unmatched groups). B) MACCE incidence, Kaplan-Meier curves (matched groups). TAVI=transcatheter aortic valve implantation.

Patients undergoing TAVI showed a higher cumulative incidence of MACCEs at 36 months (unmatched: SUAVR 17.2%, 95% CI 10.4-21.2 *vs.* TAVI 29.4%, 95% CI 22.0-36.2, *P*<0.001; matched: SUAVR 18.1%, 95% CI 10.1-25.6 vs. TAVI 32.6%, 95% CI 26.0-48.1, *P*<0.001) (Figure 4A-B).

At multivariable Cox regression analysis, TAVI was identified as an independent predictor for MACCEs (HR 2.65, 95% CI 1.26-3.86, *P*=0.003).

## DISCUSSION

To our knowledge, this is the first multi-institutional study comparing the hemodynamic performances of sutureless aortic valves *vs.* TAVI in patients with a SAA. Although not being randomized, this retrospective analysis was designed as propensity matched comparison to reduce confounding factors.

The major findings of this study were: 1) SUAVR and TAVI showed up to one year comparable hemodynamic performances in terms of iEOA, transvalvular gradients, and incidence of moderate to severe PPM; 2) at three years, patients treated with TAVI showed a significant reduction of iEOA with a significant higher rate of moderate to severe PPM when compared to SUAVR; 3) at one and three years, TAVI group showed a higher all-cause mortality when compared to SUAVR, significantly in the unmatched population; 4) at one and three years, TAVI group showed a significantly higher rate of MACCEs in the matched and unmatched groups; 5) multivariable Cox regression analysis showed PPM and PMI as independent predictors of mortality (matched PPM: HR 2.72, 95% CI 1.25-5.94, *P*=0.012) (matched: PMI: HR 5.2, 95% CI 2.0-14.3, *P*=0.012). The current study analyzed the influence of treatment strategy in patients with AS and SAA for which the risk of suboptimal valve hemodynamics and PPM is relevant.

PPM is a well-known condition which may occur in patients with SAA both after surgical AVR and TAVI procedures. Small size of stented bioprostheses (≤ 21 mm) in surgical AVR likely leads to PPM in patients with body surface area (BSA) > 1.7^[1,13]^. The risk of PPM may be reduced by using annulus enlargement techniques, allowing the use of larger bioprostheses^[14]^. However, annulus enlargement is seldom performed^[7]^, as reported by Pibarot et al., which found in the Placement of AoRTic TraNscathetER Valves (PARTNER) cohort A analysis, that patients undergoing surgical AVR had a significantly higher incidence of moderate and severe PPM when compared to TAVI. In addition, in patients with a SAA treated with stented bioprosthesis, severe PPM were found in more than one-third of cases (34%), clearly indicating a suboptimal surgical treatment^[7]^.

However, the issue of PPM remains relevant even in patients undergoing transcatheter valve implantation, since Pibarot et al., in the PARTNER trial Cohort-A analysis, reported in the subset TAVI group with SAA an incidence of moderate and severe PPM of 27% and 20%, respectively^[7]^.

Herrmann et al.^[15]^, analyzing data on more than 60,000 patients undergoing TAVI from the STS/ACC TVT Registry™, reported an incidence of moderate and severe PPM of 25% and 12%, respectively. These authors showed that PPM was associated with a significant higher mortality and rehospitalization for heart failure at one year postoperatively^[15]^. Data from TAVI Registries (International Multicenter Registry to Evaluate the Performance of Self-Expandable Valves in Small Aortic Annuli [TAVI-SMALL], Optimized transCathEter vAlvular interventioN-Transcatheter Aortic Valve Implantation [OCEAN-TAVI]), reporting outcomes in SAA patients, are consistent with the results of the abovementioned study^[8,16]^. Results of the present study confirm these findings and confirm the progressive decrease of the iEOA over time in the TAVI group, with a constant increase of moderate to severe PPM incidence^[16]^.

Progressive reduction of the iEOA may be due to an early degeneration process of TAVI caused by leaflet stress, and/or to a progressive degeneration and calcification of the “left-in-place” native valve^[17]^. Moderate and severe structural valve deterioration at mid-term in TAVIs have been reported up to 10.8% and 12.9%, respectively^[18]^.

Sutureless aortic valves were designed to overcome the major hemodynamic drawbacks of stented bioprostheses. The absence of an outer stent provides a greater EOA with a significant lower incidence of PPM when compared to stented valves^[18,19,20]^. On this regard, Tasca et al. demonstrated in small valve sizes (sutureless small and medium) an *in-vitro* hemodynamic performance as those of native aortic valves. These data are consistent with the outcomes reported by Shalabi and Rubino^[18,21]^ that showed in patients with SAA a postoperative mean iEOA of 1.12 cm^2^/m^2^ at rest^[19]^, with an increment of 30% of iEOA during stress echocardiography^[21]^. However, some technical pitfalls, such as sutureless oversizing and an incomplete annular decalcification, may jeopardize SUAVR hemodynamics, increasing the risk of valve dysfunction and PPM as reported by Belluschi and Glauber^[20,22]^.

One of the main findings of the current study is a stable and reliable hemodynamic performance of SUAVR without a significant iEOA reduction over time, avoiding the late development of PPM. Meuris et al. analyzing a large series of sutureless AVR demonstrated a survival freedom from structural valve degeneration of 97% at 10 years^[23]^.

Of note, in this study, the presence of moderate to severe PPM increased 2.5-fold the risk of mortality at follow-up. Similarly, Pibarot et al. reported in 2006 a two-fold and an 11-fold incremented risk of mortality for patients with moderate and severe PPM, respectively^[1]^. An additional important finding of the present study is the incidence of moderate to severe PVL significantly higher in the TAVI cohort when compared to the SUAVR group (6.8% *vs.* 1.4%, respectively), which is consistent with previous studies^[3,8,9]^ and TAVI registries (OCEAN-TAVI, PARTNER II)^[9,24]^, showing that moderate to severe PVL increases over the years, particularly in BEV. This is associated with a significant decrease in survival at two years^[24]^.

This study showed significantly higher rates of mortality in the TAVI group (6.1%) when compared to the SUAVR group (1.4%) at 30 days. The early mortality rate of the TAVI group could be explained by the higher percentage of patients undergoing TA procedures in this study (26.0%), higher than those in PARTNER II and Surgical Replacement and Transcatheter Aortic Valve Implantation (or SURTAVI) (17.2% and 0%, respectively). However, mortality of the TAVI group at one year and three years was significantly higher than SUAVR only in the unmatched group (Figure 3). These outcomes on TAVI patients are consistent with results reported in the OCEANTAVI registry and TAVI-SMALL at 12 and 36 months^[9]^. It should be remembered that these patients had a lower BSA than the average population (< 1.60 m^2^ in the matched group), meaning, besides a smaller aortic annulus, smaller vascular accesses^[25]^. Consequently, transvessel approaches were either not always viable or carried an elevated risk of vascular complications, making the TA approach the most feasible option.

A relevant finding of the current study is the incidence of AV blocks and left bundle branch block requiring permanent PMI that was significantly higher in the TAVI group than in the SUAVR group (11.5% *vs.* 4.5%, respectively). Those results are consistent with data from the OCEAN-TAVI and TAVI-SMALL registries (13.3% and 15.6%, respectively), while data concerning SUAVR are comparable to those reported in literature^[18]^. The lower incidence of PMI in SUAVR, which basically has the same expandable self-anchoring stent of TAVI, may be explained by the removal in SUAVR of the native aortic valves and annular calcification, which may reduce the compression causing injury to the conduction tissue^[22]^. At multivariable Cox regression analysis of the overall study population, the PMI implantation was an important predictor of mortality with a three-fold increased risk of death at three years (HR: 3.05, 95% CI 1.34-6.94).

### Limitations

The major limitation of the current study is the lack of randomization. This could be only partially corrected by propensity score matching, which reduced the heterogeneity between groups, but could not eliminate enrollment biases. However, enrollment biases may be also present in randomized comparisons when selection at the entry point of the studies takes only a small percentage of patients having the inclusion criteria.

## CONCLUSION

In conclusion, this study showed that postoperative hemodynamic performances of TAVI *vs.* SUAVR are comparable up to one year postoperatively. However, TAVI patients showed a decline in hemodynamic performance and an increase in PPM at three years, suggesting early device degeneration.

TAVI patients are burdened over time by an increased incidence of moderate to severe PVL and by higher rates of permanent PMI. PPM and PMI were associated with a significant reduction in survival both in SUAVR and TAVI groups.

In patients with AS and SAA, sutureless bioprostheses significantly improved hemodynamics and MACCEs at three years when compared to TAVI.

## Abbreviations, Acronyms & Symbols

AS: Aortic stenosis
AV: Atrioventricular
AVR: Aortic valve replacement
BEV: Balloon-expandable valves
BMI: Body mass index
BSA: Body surface area
CAD: Coronary artery disease
CI: Confidence interval
COPD: Chronic obstructive pulmonary disease
CPB: Cardiopulmonary bypass
CVA: Cerebrovascular accident
OCEAN-TAVI: Optimized transCathEter vAlvular interventioN-Transcatheter Aortic Valve Implantation
PAD: Peripheral artery disease
PARTNER: Placement of AoRTic TraNscathetER Valves
PM: Pacemaker
PMI: Pacemaker implantation
PPM: Patient-prosthesis mismatch
PVL: Perivalvular leak
SAA: Small aortic annulus
SB: Stentless bioprostheses
SD: Standard deviation
EOA: Effective orifice area
EuroSCORE: European System for Cardiac Operative Risk Evaluation
EF: Ejection fraction
Gmax: Maximum gradient
Gmean: Mean gradient
HR: Hazar ratio
HTN: Hypertension
ICU: Intensive care unit
IQR: Interquartile range
iEOA: Indexed effective orifice area
MACCEs: Major adverse cardiovascular and cerebrovascular events
MAV: Mechanical invasive ventilation
SEV: Self-expandable valves
STS: Society of Thoracic Surgeons
SUAVR: Sutureless aortic valve replacement
TA: Transapical
TAVI: Transcatheter aortic valve implantation
TAVI-SMALL: International Multicenter Registry to Evaluate the Performance of Self-Expandable Valves in Small Aortic Annuli
TF: Transfemoral
TIA: Transitory ischemic attack
VARC: Valve Academic Research Consortium

## References

[1] Fallon JM, DeSimone JP, Brennan JM, O'Brien S, Thibault DP, DiScipio AW (2018). The incidence and consequence of prosthesis-patient mismatch
after surgical aortic valve replacement. Ann Thorac Surg.

[2] Bleiziffer S, Eichinger WB, Hettich I, Guenzinger R, Ruzicka D, Bauernschmitt R (2007). Prediction of valve prosthesis-patient mismatch prior to aortic
valve replacement: which is the best method?. Heart.

[3] Guimarães L, Voisine P, Mohammadi S, Kalavrouzioutis D, Dumont E, Doyle D (2020). Valve hemodynamics following transcatheter or surgical aortic
valve replacement in patients with small aortic annulus. Am J Cardiol.

[4] Penaranda JG, Greason KL, Pislaru SV, Schaff HV, Daly RC, Park SJ (2014). Aortic root enlargement in octogenarian patients results in less
patient prosthesis mismatch. Ann Thorac Surg.

[5] Cheng D, Pepper J, Martin J, Stanbridge R, Ferdinand FD, Jamieson WR (2009). Stentless versus stented bioprosthetic aortic valves: a
systematic review and meta-analysis of controlled trials. Innovations (Phila).

[6] Harky A, Wong CHM, Hof A, Froghi S, Ahmad MU, Howard C (2018). Stented versus stentless aortic valve replacement in patients
with small aortic root: a systematic review and
meta-analysis. Innovations (Phila).

[7] Pibarot P, Weissman NJ, Stewart WJ, Hahn RT, Lindman BR, McAndrew T (2014). Incidence and sequelae of prosthesis-patient mismatch in
transcatheter versus surgical valve replacement in high-risk patients with
severe aortic stenosis: a PARTNER trial cohort--a analysis. J Am Coll Cardiol.

[8] Regazzoli D, Chiarito M, Cannata F, Pagnesi M, Miura M, Ziviello F (2020). Transcatheter self-expandable valve implantation for aortic
stenosis in small aortic annuli: the TAVI-SMALL registry. JACC Cardiovasc Interv.

[9] Abdelghani M, Mankerious N, Allali A, Landt M, Kaur J, Sulimov DS (2018). Bioprosthetic valve performance after transcatheter aortic valve
replacement with self-expanding versus balloon-expandable valves in large
versus small aortic valve annuli: insights from the CHOICE trial and the
CHOICE-extend registry. JACC Cardiovasc Interv.

[10] Mauri V, Kim WK, Abumayyaleh M, Walther T, Moellmann H, Schaefer U (2017). Short-term outcome and hemodynamic performance of next-generation
self-expanding versus balloon-expandable transcatheter aortic valves in
patients with small aortic annulus: a multicenter propensity-matched
comparison. Circ Cardiovasc Interv.

[11] Meco M, Montisci A, Miceli A, Panisi P, Donatelli F, Cirri S (2018). Sutureless perceval aortic valve versus conventional stented
bioprostheses: meta-analysis of postoperative and midterm results in
isolated aortic valve replacement. J Am Heart Assoc.

[12] Généreux P, Piazza N, Alu MC, Nazif T, Hahn RT, VARC-3 WRITING COMMITTEE: (2021). Valve academic research consortium 3: updated endpoint
definitions for aortic valve clinical research. J Am Coll Cardiol.

[13] Dayan V, Vignolo G, Soca G, Paganini JJ, Brusich D, Pibarot P (2016). Predictors and outcomes of prosthesis-patient mismatch after
aortic valve replacement. JACC Cardiovasc Imaging.

[14] Ghoneim A, Bouhout I, Demers P, Mazine A, Francispillai M, El-Hamamsy I (2016). Management of small aortic annulus in the era of sutureless
valves: a comparative study among different biological
options. J Thorac Cardiovasc Surg.

[15] Herrmann HC, Daneshvar SA, Fonarow GC, Stebbins A, Vemulapalli S, Desai ND (2018). Prosthesis-patient mismatch in patients undergoing transcatheter
aortic valve replacement: from the STS/ACC TVT registry. J Am Coll Cardiol.

[16] Hase H, Yoshijima N, Yanagisawa R, Tanaka M, Tsuruta H, Shimizu H (2021). Transcatheter aortic valve replacement with evolut R versus
sapien 3 in Japanese patients with a small aortic annulus: the OCEAN-TAVI
registry. Catheter Cardiovasc Interv.

[17] Kataruka A, Otto CM (2018). Valve durability after transcatheter aortic valve
implantation. J Thorac Dis.

[18] Shalabi A, Spiegelstein D, Sternik L, Feinberg MS, Kogan A, Levin S (2016). Sutureless versus stented valve in aortic valve replacement in
patients with small annulus. Ann Thorac Surg.

[19] Tasca G, Vismara R, Mangini A, Romagnoni C, Contino M, Redaelli A (2017). Comparison of the performance of a sutureless bioprosthesis with
two pericardial stented valves on small annuli: an in vitro
study. Ann Thorac Surg.

[20] Belluschi I, Moriggia S, Giacomini A, Del Forno B, Di Sanzo S, Blasio A (2017). Can perceval sutureless valve reduce the rate of
patient-prosthesis mismatch?†. Eur J Cardiothorac Surg.

[21] Rubino AS, Biancari F, Caruso V, Lavanco V, Privitera F, Rinaldi I (2018). Hemodynamic assessment of perceval sutureless bioprosthesis by
dobutamine stress echocardiography. Echocardiography.

[22] Glauber M, Miceli A, di Bacco L (2020). Sutureless and rapid deployment valves: implantation technique
from A to Z-the perceval valve. Ann Cardiothorac Surg.

[23] Szecel D, Eurlings R, Rega F, Verbrugghe P, Meuris B (2021). Perceval sutureless aortic valve implantation: midterm
outcomes. Ann Thorac Surg.

[24] Leon MB, Smith CR, Mack MJ, Makkar RR, Svensson LG, Kodali SK (2016). Transcatheter or surgical aortic-valve replacement in
intermediaterisk patients. N Engl J Med.

[25] Nakashima M, Watanabe Y (2018). Transcatheter aortic valve implantation in small anatomy: patient
selection and technical challenges. Interv Cardiol.

